# Basic Biology and Clinical Application of Multipotent Mesenchymal Stromal Cells: From Bench to Bedside 

**DOI:** 10.1155/2012/185943

**Published:** 2012-08-28

**Authors:** Selim Kuçi, Reinhard Henschler, Ingo Müller, Ettore Biagi, Roland Meisel

**Affiliations:** ^1^Departments of Hematology and Oncology, University Children's Hospital, Theodor-Stern-Kai 7, 60590 Frankfurt, Germany; ^2^Institute for Transfusion Medicine, Cell Therapeutics and Hemostaseology, Ludwig Maximilians University Munich, Marchioninistrasse 15, 81377 Munich, Germany; ^3^Clinic of Pediatric Hematology and Oncology, University Medical Center Hamburg-Eppendorf, Martinistrasse 52, 20246 Hamburg, Germany; ^4^Laboratory of Cell Therapy “Stefano Verri," Pediatric Department, University of Milano-Bicocca, San Gerardo Hospital, 20900 Monza, Italy; ^5^Department of Pediatric Oncology, Hematology, and Clinical Immunology, Medical Faculty, Heinrich-Heine University Düsseldorf, 40225 Düsseldorf, Germany

Since their discovery in the end of the 1960s and the beginning of the 1970s by Alexander Friedenstein, mesenchymal stromal cells (MSCs) have attracted the interest of the scientific community because of their ubiquitous presence in the organism, the ease of their procurement, and potential to differentiate into other tissues. As a nonhematopoietic cell population, first described in the bone marrow, MSCs comprise a pool of progenitor cells with different proliferative, differentiation, immunosuppressive, and hematopoietic engraftment-promoting potential. To bring more insights into cellular and molecular mechanisms of action of MSCs and their potential for clinical use, stem cells international set out to launch and publish a special Issue on this topic. In this issue, distinguished researchers from all over the world contributed with their experience and knowledge to highlight the latest developments in this emerging field of research.

The conundrum of the MSC origin remains unresolved due to the lack of a specific cell surface marker. Identification of such markers could undoubtedly help the scientists to develop new methodologies for prospective isolation and expansion of MSCs in their “native” state (undifferentiated) for clinical application.

S. A. E. Boxall and Jones in the UK (in “*Markers for characterization of bone marrow multipotential stromal cells*”) have focused on description of the most important markers, which have been reported so far for the characterization and prospective isolation of progenitor cells for MSCs. In the first part of this paper, the authors discussed surface and molecular markers that were proposed as the indicators of MSC potency, in terms of their proliferative potential or the ability to differentiate into desired lineages. In the second part of this paper, the authors discussed critically surface markers of uncultured (i.e., native) bone marrow-(BM-) derived MSCs. Although no formal consensus has yet been reached on which markers may be best suited for prospective BM-MSC isolation, the authors suggest that markers that cross-react with MSCs of animal models (such as CD271 and W8-B2/MSCA-1) may have the strongest translational value.

As the number of progenitor cells for MSCs in BM and other organs is pretty low, MSCs should be *ex vivo* expanded in order to achieve a sufficient number for clinical application. Historically, large-scale clinical expansions of MSCs have been performed by using a basal medium supplemented with fetal bovine serum (FBS). Because of the ill-defined nature of FBS (presence of xenogeneic components) and lot-to-lot inconsistency of performance, development of a more defined serum-free MSC culture medium has become absolutely necessary. Therefore, S. Jung et al. in Canada (in “*Ex vivo expansion of human mesenchymal stem cells in defined serum-free media*”) reviewed herein current cell culture media for hMSCs and discussed medium development strategies. In addition to the serum-containing and serum-free MSC-culture medium supplemented with platelet lysates the authors compared properties of a serum-free, chemically defined MSC-culture medium (PPRF-msc6) developed in their laboratory with the commercially available serum-free media.

MSCs after their *in vivo* administration show their anti-inflammatory and regenerative effects either by secretion of long-distance acting soluble molecules or by migrating and homing to the damaged tissues. S. k. Kang et al. in South Korea in their review (in “*Journey of mesenchymal stem cells for homing: strategies to enhance efficacy and safety of stem cell therapy*”) outline the current understanding of MSC migration and discuss strategies for enhancing both the environmental and cellular conditions that give rise to effective homing of MSCs. According to the authors, this may allow MSCs to quickly find and migrate to injured tissues, where they may best exert clinical benefits resulting from improved homing and the presence of increased numbers of MSCs.

Extensive experimental research during the past decades profoundly influenced the understanding of the mechanism of MSC-action and consequently its potential use in regenerative medicine. P. Babei et al. in Iran (in “*Transplanted bone marrow mesenchymal stem cells improve memory in rat models of alzheimer's disease*”) demonstrated in the rat model that MSC treatment significantly increased learning ability and memory in both age-induced and chemically induced memory impairment.

In addition, stem cell-based therapy represents a rapidly growing alternative for cardiovascular diseases as the leading cause of death worldwide. M. T. Elnakish et al. in USA (in “*Mesenchymal stem cells for cardiac regeneration: translation to bedside reality*”) focussed on therapeutic applications of MSCs and their transition from experimental bench side to clinical bedside. Based on the reports published so far, they conclude that in addition to embryonic stem cells, induced pluripotent stem cells (iPSCs), endothelial progenitor cells, hematopoietic stem cells, and skeletal myoblasts that were used in the treatment of ischemic heart disease and myocardial infarction, MSCs due to their distinctive properties represent an innovative approach for cardiac regeneration. In line with this, N. A. Kouris et al. in USA (in “*Directed fusion of mesenchymal stem cells with cardiomyocytes via VSV-G facilitates stem cell programming*”) in their original article demonstrated that expression of the fusogen of the vesicular stomatitis virus (VSV-G) in human MSCs (vMSCs) increased their fusion capacity with cardiomyocytes (CMs). The fused cells adopted a CM-like phenotype and morphology *in vitro*. Furthermore, the authors found that the vMSCs delivered to the damaged mouse myocardium, via a collagen patch, were able to home to the myocardium and fuse to cells within the infarct and peri-infarct region of the myocardium and exerted functional benefit via multiple mechanisms.

H. Thaker and A. K. Sharma in USA (in “*Engaging stem cells for customized tendon regeneration*”) outlined in their review the use of MSCs together with poly(1,8-octanediol co-citrate) scaffolds (POCs) to develop a fibroelastic network guided by cytokines and growth factors in order to be used as a consistent therapeutic approach to tendon injury repair.

R. Nuzzi et al. in Italy (in “*Effect of in vitro exposure of corticosteroid drugs, conventionally used in AMD treatment, on mesenchymal stem cells*”) in their research article found that steroid drugs, often used to treat age-related macular degeneration (AMD), demonstrate a negative effect on MSCs as a potential candidate for treatment of AMD. This effect was reduced in the presence of supernatant of a human retinal pigment epithelial cell line (ARPE-19).

As MSCs exert a very potent immunomodulatory effect, they are often used clinically to suppress the adverse effects of graft versus host disease (GvHD) after hematopoietic stem cell transplantation. Because of a broad immunosuppression, the patients are at risk of bacterial and viral infections. G. Lucchini et al. in Italy (in “*Mesenchymal stromal cells do not increase the risk of viral reactivation nor the severity of viral events in recipients of allogeneic stem cell transplantation*”) in their clinical study analyzed viral reactivation episodes by a whole blood PCR in 24 patients receiving MSCs for the treatment of GvHD. In their cohort of patients, viral reactivation after MSC infusion occurred in 45% of the cases, which did not significantly differ from the incidence in a historical cohort of patients affected by steroid-resistant GvHD and treated with conventional immunosuppression.

In addition to the bone marrow, during the last decade the adipose tissue is being recognized not only as an energy reservoir, but also as a rich source of multipotent cells. P. C. Baer and H. Geiger in Germany (in “*Adipose-derived mesenchymal stromal/stem cells: tissue localization, characterization, and heterogeneity*”) and H. Orbay et al. in Japan (in “*Mesenchymal stem cells isolated from adipose and other tissues: basic biological properties and clinical applications*”) in their review articles made an overview about the sources and methods of isolation, phenotype as well as the potential of adipose tissue-derived MSCs to give rise to the tissues of three germ layers. In line with this, R. k. Chan et al. in USA (in “*Development of a vascularized skin construct using adipose-derived stem cells from debrided burned skin*”) have exploited autologous stem cells from the adipose layer of surgically debrided burned skin (dsASC) to generate *in vitro* an epithelial layer, a vascularised dermal layer, and a hypodermal layer. The authors conclude that this technique may provide an alternative approach for cutaneous coverage after extensive burn injuries.

Regulatory issues concerning the safety of clinical use of MSCs are strongly required for their broad clinical application. Y. Wang et al. in China (in “*Safety of mesenchymal stem cells for clinical application*”) in their review focussed on safety issues of MSCs, in particular their genetic stability in long-term *in vitro* expansion, their cryopreservation, banking, and the role of serum in the preparation of MSCs. Based on hundreds of clinical trials using MSCs that have been registered, the authors conclude that the production of safe cell products requires an entire process of supervision in order to assure that the cells maintain overall phenotype, functional potential, and to ensure that cultured cells remain untransformed and without microbiological contaminations.

In summary, we hope that the reader of this special issue will gain more insights into the advancements and challenges faced by this rapidly expanding field of medicine.

*Selim Kuçi* *Selim Kuçi* *Reinhard Henschler* *Reinhard Henschler* *Ingo Müller* *Ingo Müller* *Ettore Biagi* *Ettore Biagi* *Roland Meisel* *Roland Meisel*

